# Early Invasion of Brain Parenchyma by African Trypanosomes

**DOI:** 10.1371/journal.pone.0043913

**Published:** 2012-08-31

**Authors:** Ute Frevert, Alexandru Movila, Olga V. Nikolskaia, Jayne Raper, Zachary B. Mackey, Maha Abdulla, James McKerrow, Dennis J. Grab

**Affiliations:** 1 Division of Medical Parasitology, Department of Microbiology, New York University School of Medicine, New York, New York, United States of America; 2 Department of Pathology, The Johns Hopkins University School of Medicine, Baltimore, Maryland, United States of America; 3 Department of Pediatrics, The Johns Hopkins University School of Medicine, Baltimore, Maryland, United States of America; 4 Department of Biological Sciences, Hunter College of CUNY, New York, New York, United States of America; 5 Department of Pathology, University of California San Francisco, San Francisco, California, United States of America; Royal Tropical Institute, The Netherlands

## Abstract

Human African trypanosomiasis or sleeping sickness is a vector-borne parasitic disease that has a major impact on human health and welfare in sub-Saharan countries. Based mostly on data from animal models, it is currently thought that trypanosome entry into the brain occurs by initial infection of the choroid plexus and the circumventricular organs followed days to weeks later by entry into the brain parenchyma. However, *Trypanosoma brucei* bloodstream forms rapidly cross human brain microvascular endothelial cells *in vitro* and appear to be able to enter the murine brain without inflicting cerebral injury. Using a murine model and intravital brain imaging, we show that bloodstream forms of *T. b. brucei* and *T. b. rhodesiense* enter the brain parenchyma within hours, before a significant level of microvascular inflammation is detectable. Extravascular bloodstream forms were viable as indicated by motility and cell division, and remained detectable for at least 3 days post infection suggesting the potential for parasite survival in the brain parenchyma. Vascular inflammation, as reflected by leukocyte recruitment and emigration from cortical microvessels, became apparent only with increasing parasitemia at later stages of the infection, but was not associated with neurological signs. Extravascular trypanosomes were predominantly associated with postcapillary venules suggesting that early brain infection occurs by parasite passage across the neuroimmunological blood brain barrier. Thus, trypanosomes can invade the murine brain parenchyma during the early stages of the disease before meningoencephalitis is fully established. Whether individual trypanosomes can act alone or require the interaction from a quorum of parasites remains to be shown. The significance of these findings for disease development is now testable.

## Introduction

African trypanosomes replicate at the tsetse fly bite site before migrating from the skin via the lymphatic system into the bloodstream to infect the spleen, liver, lymph nodes, skin, heart, eyes, and endocrine system [1], [2]. If untreated during the early stage (Stage-1), the parasites later produce central nervous system (CNS) disease (Stage-2), a process that takes months to years with *T. b. gambiense* (West African) and weeks to months with *T. b. rhodesiense* (East African) sleeping sickness. Human African trypanosomiasis (HAT) associated meningoencephalitis leads to progressive neurologic involvement with concomitant neuropsychiatric disorders, fragmentation of the circadian sleep-wake cycle, and eventually death [1]–[3]. Once Stage-2 CNS disease is established, the parasites are shielded from the many trypanocidal drugs and the brain may be a source for relapse [4], [5].

Longitudinal studies in mice all describe one general progression pattern of late-stage brain histopathology [6]–[14]. Trypanosomes initially accumulate in the stroma of the highly vascularized choroid plexus, between a layer of fenestrated endothelia and a layer of epithelia that produce cerebrospinal fluid (CSF). The resulting inflammatory response disseminates from the choroid plexus via the microvasculature into the parenchyma in three characteristic phases: leptomeningitis, early meningoencephalitis, and encephalitis. According to the current paradigm, trypanosomes invade the brain parenchyma only at the final stages of the disease, when meningoencephalitis is fully established. Both histopathology and composition of the inflammatory infiltrate observed in the murine model mimic autopsy findings in HAT patients [11], [15]–[18].

African trypanosomes (*T. brucei* spp.) upregulate ICAM-1, VCAM-1 and E-selectin expression in brain and non-brain endothelial cells [19], [20]. Our studies using human brain microvascular endothelial cells (HBMEC) as a blood brain barrier (BBB) model show a functional link between trypanosome cysteine proteases, Gαq-mediated calcium signaling, and protease-activated receptor-2 (PAR-2) in BBB transmigration [21]–[24]. Increased chemokine/cytokine protein expression with trypanosome infection and gene-profiling data identified several candidate pathways as links between brain inflammatory processes and CNS HAT [1], [24], [25].

Based on these findings, we hypothesized that African trypanosomes, either individually or in groups, have the potential for rapid BBB traversal *in vivo*. The following intravital microscopy (IVM) study of the murine cortical microvasculature supports this hypothesis by showing early BBB crossing and parasites in the brain parenchyma within hours after entering the bloodstream.

## Results

Our studies on the role of cysteine proteases in the pathogenesis of *T. b. brucei* 90-13 trypanosomiasis, both in a mouse model and with in an *in vitro* model of the BBB, strongly implicated brucipain in facilitating trypanosome entry into the brain [26]. To verify the parasites’ potential for CNS entry, mice received inocula of 10^5^ or 10^6^ *Tbb*-O BSF to increase the likelihood of parasite detection in the brain by IVM. Assuming a total blood volume of 1.6 mL in a 23 g C57Bl6 mouse, an inoculum of 10^5^ and 10^6^ *Tbb*-O should result in a respective parasitemia of 6.3×10^4^ and 6.3×10^5^ parasites/mL immediately post infection, numbers that exceed the initial parasitemia generated by the bite of a single infected Tsetse fly, but are typically reached in natural infections of livestock animals (*T. b. brucei*) or humans (*T. b. rhodesiense*). Other than the expected lower parasite numbers observed after administration of 10^5^ trypanosomes, IVM revealed no major differences between the two inoculation regimens, in particular during the first hours after infection.

### Day 1 Post Infection: *T. b. brucei* 90-13 Enters the Brain Parenchyma

Mice infected intravenously with 10^6^ *Tbb*-O BSF were subjected to craniotomy and immobilized on the stage of an inverted confocal microscope for intravital imaging of the cerebral cortex [27]. Nuclei and the vascular lumen were visualized by intravenous inoculation of Hoechst 33342 and BSA-Alexa 647, respectively. Capillaries and postcapillary venules (PCVs), the morphological correlates of the physiological and neuroimmunological BBB [28], were identified by their respective diameters of ≤6 µm and 20–60 µm [29]. PCVs were distinguished from similarly sized arterioles by the direction of the blood flow [27]. Mice whose meningeal vasculature was injured during craniotomy exhibited extensive bleeding and were excluded from the study. None of the mice used for this study exhibited any evidence for neurological impairment prior to surgery and imaging [30].

**Figure 1:**
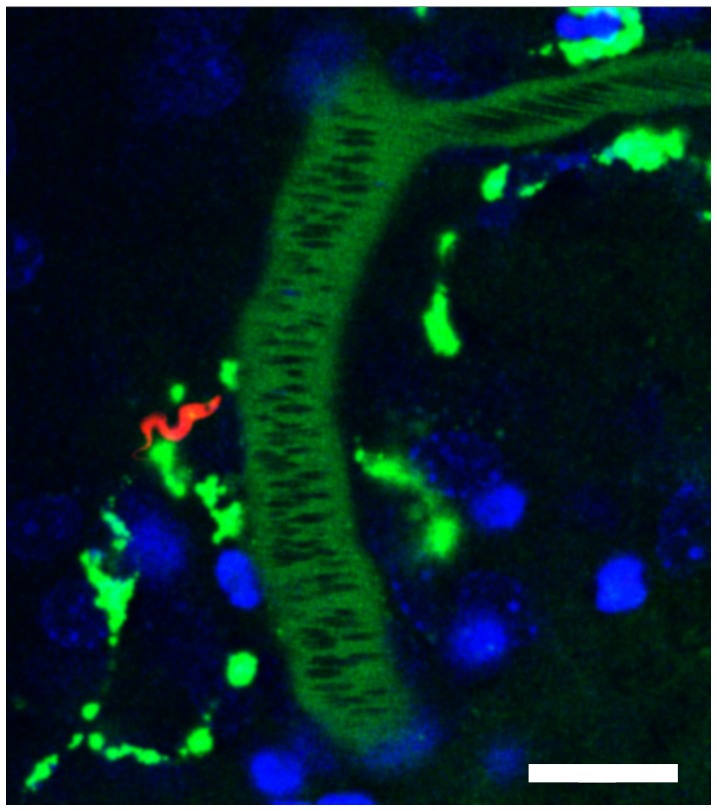
Trypanosomes enter the brain parenchyma. One day post infection with 10^6^ *Tbb-O*, mice were subjected to craniotomy and examined by confocal microscopy. The vascular lumen was visualized by intravenous inoculation of Alexa 647-conjugated BSA (shown in green). A motile *Tbb-O* BSF (red) is located adjacent to a PCV. RBCs in the lumen of the PCV exclude the vascular maker and appear as negatively stained (dark) streaks. Nuclei were stained with Hoechst (blue). Bar = 20 µm. **Movie S1**.

**Figure 2:**
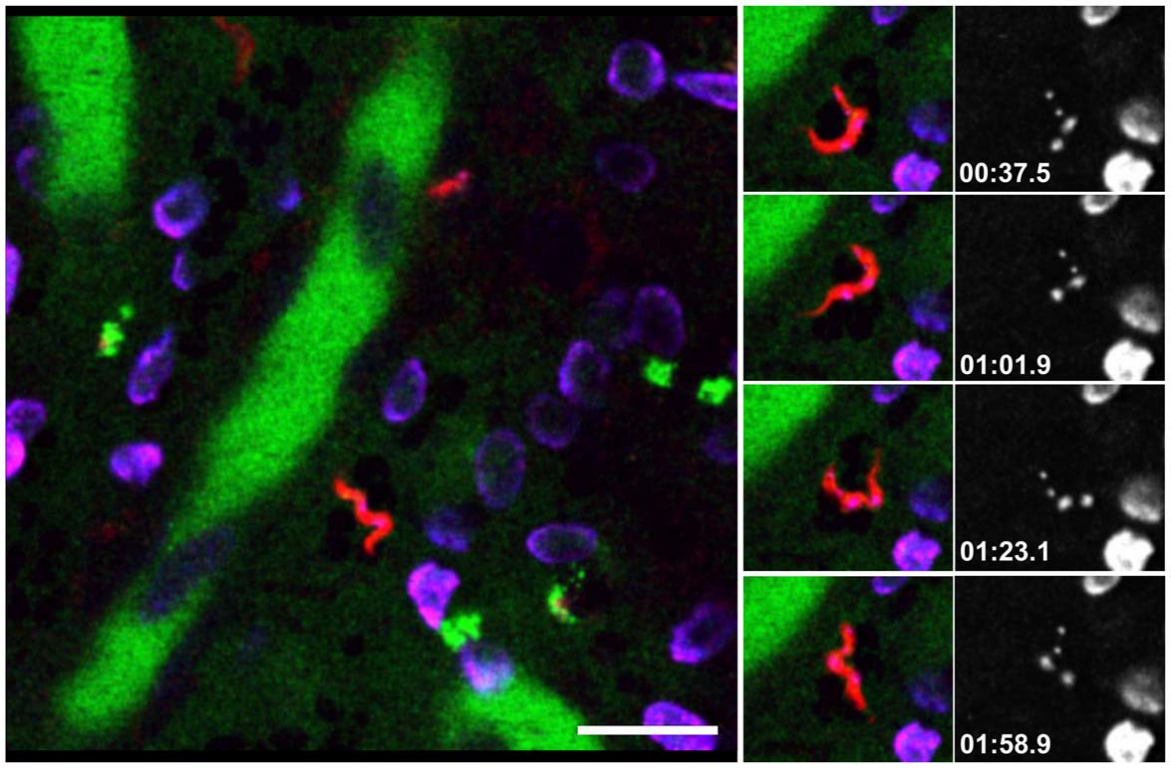
Trypanosomes divide within the brain parenchyma. DNA staining reveals two nuclei (large Hoechst-stained organelles) and two kinetoplasts (small Hoechst-stained organelles) within a motile extravascular BSF (red). The figure shows individual frames from an intravital movie recorded 24 h post infection with 10^6^ *Tbb-O*. The vascular lumen is green, nuclei are blue. The panels on the right show the two nuclei and two kinetoplasts of the dividing parasite in the blue channel. Bars = 20 µm. **Movie S2**.

**Figure 3:**
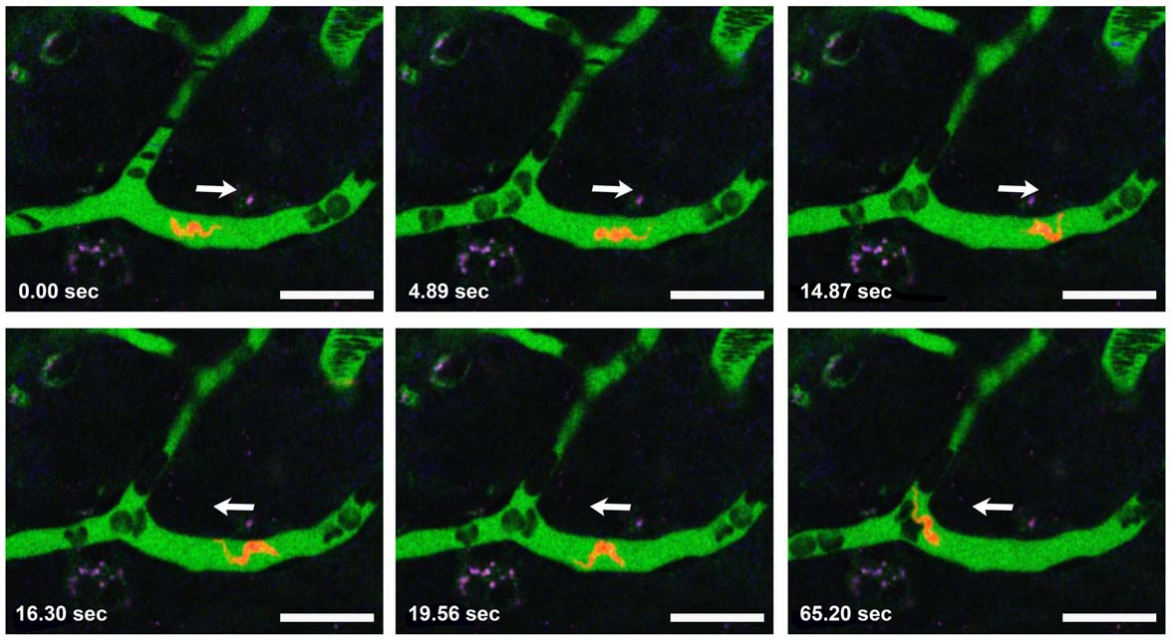
Trypanosomes travel with the flagellar tip leading. Temporary leukocyte-mediated interruption of the blood flow allows a parasite to swim freely in a capillary. Note that the negatively stained blood cells (dark) in the vascular lumen become distorted to streaks with increasing velocity. Two days post infection with 10^6^ *Tbb-O* BSF. Bars = 20 µm. **Movie S3**.

Although only low numbers of parasites were observed traveling with the bloodstream, motile *Tbb*-O BSF were clearly present in the brain parenchyma 24 h post infection (**Fig. 1**, **Movie S1**). Occasionally, a trypanosome in process of cell division was seen as indicated by the presence of two nuclei and two kinetoplasts (**Fig. 2**, **Movie S2**). In contrast to uninfected control animals, *Tbb*-O infected mice exhibited individual adherent mononuclear leukocytes in the cortical microvasculature. These leukocytes arrested in PCVs and capillaries, the latter of which they occasionally blocked. Temporary interruption of the blood flow in a capillary revealed that BSF move with the flagella tip leading (**Fig. 3**, **Movie S3**).

**Figure 4:**
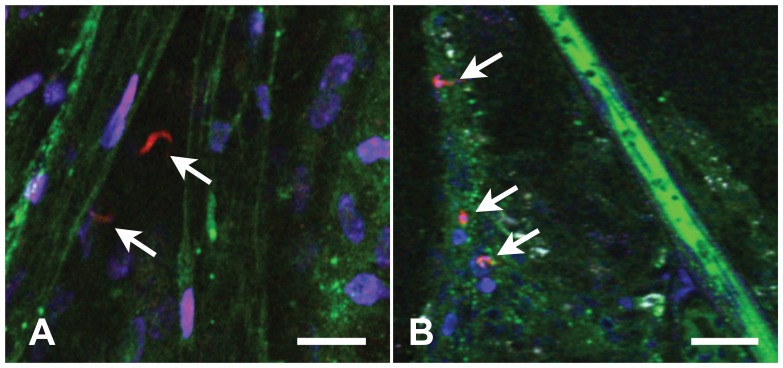
Brain sections reveal extravascular trypanosomes after low-dose infection. Screening of large areas of brain tissue was required to detect extravascular (A, arrow) and intravascular (B, arrow) *Tbb-O* (red) 24 h after infection with 10^5^ BSF. Infected mice were injected with BSA-Alexa 647 (green) and the nuclear stain Hoechst (blue) prior to removal of the brain. Immediately after vibratome sectioning, 100 µm slices of live brain tissue were subjected to *ex vivo* imaging. Traces of the vascular marker remain visible after sectioning and outline the microvascular lumen. Bars = 20 µm.

When the lower dose of 10^5^ *Tbb*-O was inoculated intraperitoneally, intravascular parasites were rare and extravascular parasites were undetectable by IVM because of constraints dictated by the field of view through a small cranial window (data not shown). The use of Vibratome sections, a technique often used for neuron imaging or electrophysiological recording of neurons especially when input-output fibers are also preserved, allowed examination of a much larger area of tissue than accessible for IVM through a small cranial window. By screening multiple vibratome sections of live brain tissue we did find parasites in the brain parenchyma (**Fig. 4**).

### Day 2 Post Infection: *T. b. brucei* 90-13 Induces Leukocyte Recruitment, Vascular Leakage, and Capillary Occlusion

**Figure 5:**
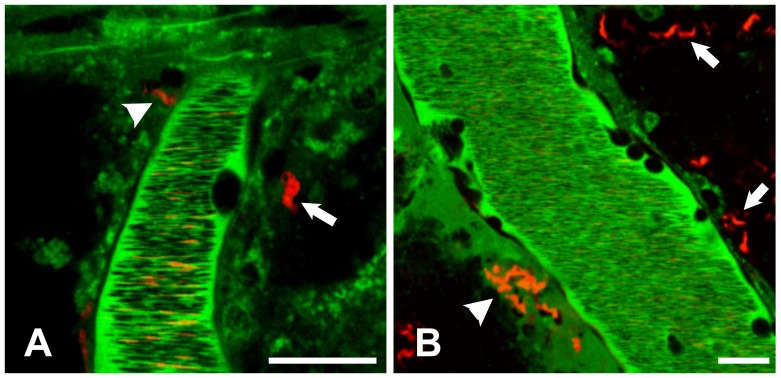
Leukocyte recruitment and vascular leakage. Two days post infection with 10^6^ *Tbb-O* BSF, the number of both intra- and extravascular trypanosomes has increased. A) Motile BSF are located in the PVS (arrowhead) surrounding a PCV and in the parenchyma (arrows). B) A cluster of extravascular BSF can be seen in a PVS (arrowhead), while numerous others have entered the parenchyma (arrows). The vascular marker BSA-Alexa 647 (shown in green) has leaked into the PVS. The PCVs contain arrested leukocytes (★). The narrow dark streaks in the PCV represent fast-moving blood cells and demonstrate unimpaired blood flow. Bars = 20 µm. **Movie S4 and S5.**

At 48 h after infection, IVM of mice infected intravenously with 10^6^ *Tbb*-O revealed large numbers of BSF traveling at bloodstream velocity in the cortical microvasculature and many motile BSF were found in the brain parenchyma and within the perivascular space (PVS) surrounding PCVs (**Fig. 5**, **Movie S4 and S5**). The brain also exhibited signs of microvascular inflammation: recruitment of leukocytes to PCVs, leakage of the fluorescent plasma marker into the PVS and surrounding parenchyma, and focal capillary occlusion.

**Figure 6:**
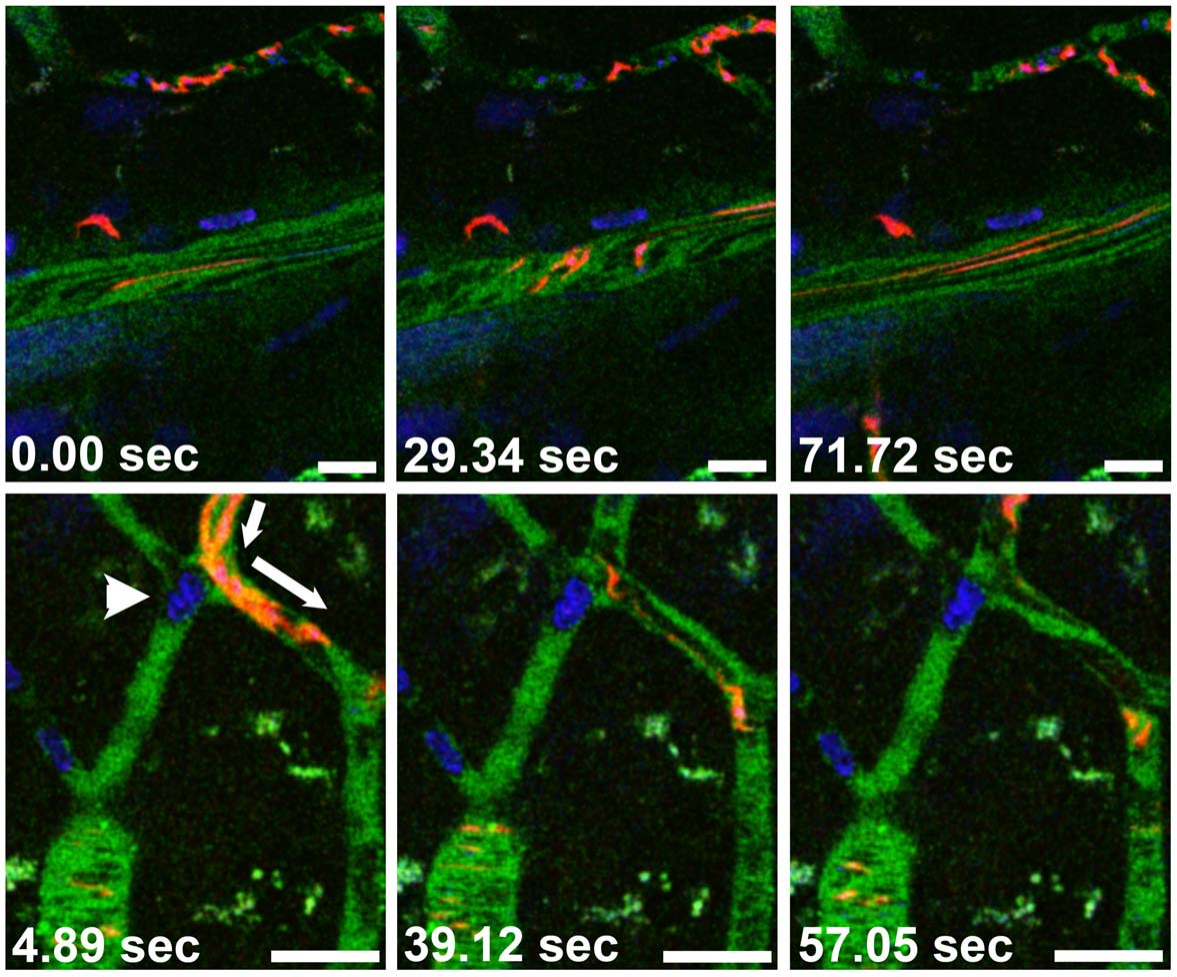
Leukocyte recruitment and vascular occlusion. Upper panel: Two days after infection with 10^5^ *Tbb-O*, a motile BSF (red) is located in the brain parenchyma near a PCV. While numerous other trypanosomes travel at bloodstream velocity in the PCV, the flow in a neighboring capillary (top) is impaired by arrested leukocytes. Lower panel: An arrested mononuclear leukocyte occludes a capillary (arrowhead) and diverts the blood flow into a collateral microvessel (arrow). Some *Tbb-O* (red) appear distorted due to their high velocity. Bars = 20 µm. **Movie S6 and S7**.

**Figure 7:**
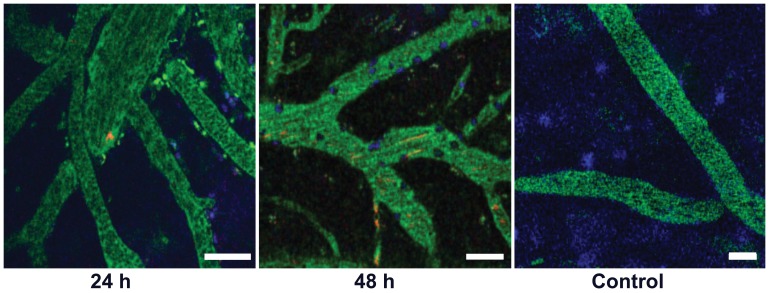
Leukocyte recruitment to PCVs. The number of arrested mononuclear leukocytes increases between 24 h and 48 h post infection with 10^5^ *Tbb-O*. In contrast, arrested cells are absent from the cortical microvasculature of an uninfected control mouse. Whereas intravascular *Tbb-O* are rare at 24 h, multiple red streaks in the vascular lumen indicate the increase in parasitemia at 48 h post infection. Bars = 20 µm. **Movie S8 and S9**.

Similarly, after intraperitoneal inoculation of 10^5^ *Tbb*-O, the number of intravascular BSF had increased relative to the previous day and extravascular parasites were detected more frequently (**Fig. 6**, **Movie S6 and S7**). IVM also revealed a few arrested mononuclear leukocytes along the wall of PCV and capillaries, but vascular leakage was less prominent 48 h after infection with 10^5^ compared to 10^6^ parasites (**Fig. 7**, **Movie S8 and S9**). Based on size, shape of nucleus, and amoeboid movement, these mononuclear cells were most likely monocytes.

### Five Hours Post Infection: *T. b. rhodesiense* IL 1852 and *T. b. brucei* GVR/35 Enter the Brain Parenchyma

**Figure 8:**
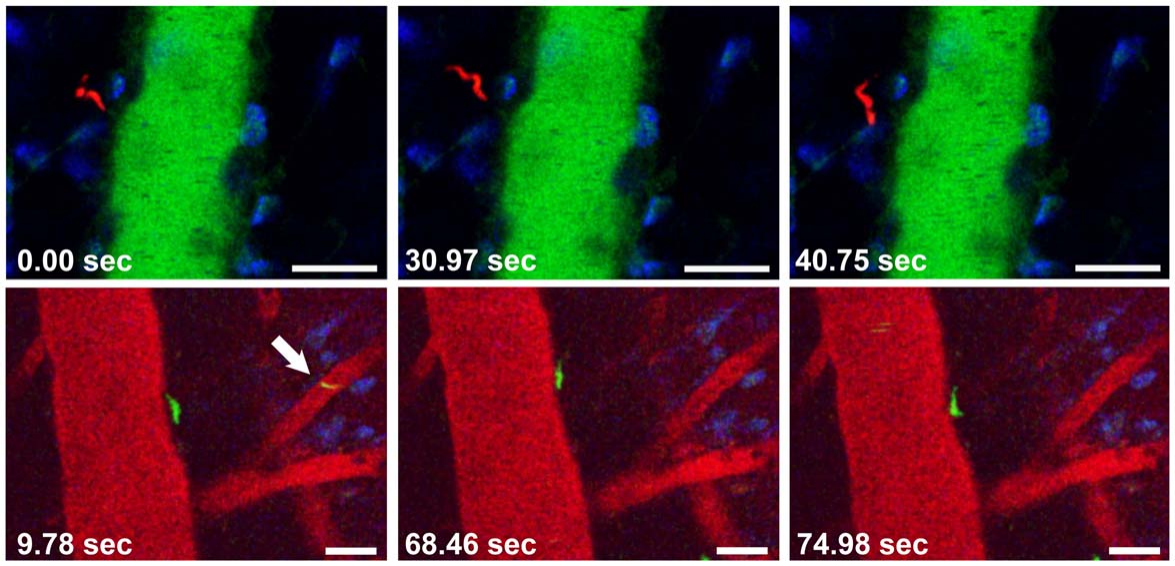
Trypanosomes enter the brain parenchyma within hours. Selected frames from intravital movies show motile BSF in the brain parenchyma 5 h after infection with 10^6^ BSF. Note the absence of arrested leukocytes and leakage of the vascular marker into the surrounding tissue. Upper panel: A motile *Tbr*-T BSF (red) is located in the cerebral parenchyma adjacent to a PCV. Lower panel: A motile *Tbb* GVR/35-PKH67 BSF (green) can be seen next to a PCV. Intravascular parasites (arrow) are rare. The vascular lumen is red, nuclei are blue. Bars = 20 µm. **Movie S10 and S11**.

Finally, we tested the potential for BBB traversal of the human CSF isolate *T. b. rhodesiense* IL1852 and the *T. b. brucei*-GVR/35 chronic mouse model trypanosome. Five hours after intravenous inoculation of 10^6^ parasites, motile *Tbr*-T BSF (**Fig. 8**, **Movie S10**) and *Tbb* GVR/35-PKH67 (**Fig. 8**, **Movie S11**) were found in the cortical microvasculature and brain parenchyma of the infected mice. Importantly, arrested leukocytes were rare and vascular leakage was not evident at this early stage of the infection.

### Location of Extravascular Trypanosomes

Determination of the location of all extravascular trypanosomes detected in this study (N = 44) with respect to the cortical microvascular tree revealed that the overwhelming majority of the BSF was associated with PCVs (66%) or similar-sized vessels (7%), few with arterioles (9%), and none with capillaries. The remaining parasites (18%) had moved too far into the parenchyma to be classified. The preferential localization of the parasites near PCV validates previous reports concerning the site of trypanosome entry into the brain [2] and confirms that parasite entry into the brain parenchyma is not simply a surgery-induced artifact.

## Discussion

IVM allowed us to visualize real time events at the level of the neurovascular unit (NVU) that occur near the surface of the cerebral cortex. Nonetheless, with its dense microvasculature, study of this region of the brain provides a valid snapshot as to what may occur in other brain areas. It is accepted that homeostatic interactions between the cellular components of the NVU (BBB endothelium, astroglia, neurons) are required for maintaining normal brain function [31]–[34]. Furthermore, links between NVU deregulation and CNS disease have been suggested for multiple sclerosis, Alzheimer’s disease, stroke, certain depression disorders, and microbial infections [35]–[38]. With the cells of the NVU being rarely located more than 20–40 µm from a microvessel, a 15–20 µm trypanosome does not have to travel far to invade the NVU and infiltrate the brain parenchyma. Nevertheless, although the mechanism of trypanosome passage across neuroimmunological BBB is likely the same throughout the brain, the morphological correlate of the NVU in the cortex likely differs from that in other areas of the brain so that the response of the parenchyma may be focally distinct. For example, selective sensitivity of the sleep centers to trypanosome infection would explain the characteristic clinical signs and pathology of HAT. Further, iNOS activation was reported to increase throughout the infection process in certain central compartments of the brain, in particular the thalamus and hypothalamus where the sleep/wake regulation is located [39], [40]. Therefore, preferential death and disintegration of trypanosomes in these areas with release of toxins and inflammatory mediators together with the cytotoxic effect of NO and its derivatives would cause neuronal injury and altered signaling and thus contribute to the typical pathogenesis of HAT [12]. Conversely, areas of low iNOS expression and NO production such as cortex may support parasite survival.

The murine model supports our hypothesis that the parasites are able to invade the CNS shortly after entering the bloodstream. Using fluorescent BSA to define the luminal boundary of brain microvascular endothelial cells that measure only 0.2–0.3 µm in thickness [41], [42], it was not difficult to define a 5×20 µm BSF trypanosome on the abluminal side of the BBB endothelium. Both *T. b. brucei* and *T. b. rhodesiense* BSF gained access to the brain parenchyma within hours after infection, before a significant level of microvascular inflammation was detectable. Extravascular BSF were viable as indicated by motility and cell division and predominantly associated with PCVs suggesting that early brain infection occurs by parasite passage across the neuroimmunological BBB. Because we did not pre-label the endothelial cells or their tight junctions, our studies are not able to determine parasites in the active act of paracellular crossing.

From our studies we cannot with certainty decipher whether trypanosomes act alone or require communication amongst a quorum of parasites to enter the brain. If individual trypanosomes are capable of early brain entry, our finding challenges the current paradigm that trypanosomes cross the murine BBB only at the onset of neurological signs, i.e. days after infection with *T. b. brucei* or *T. b. rhodesiense* and several weeks after infection with *T. b. gambiense* [43], at a time when the choroid plexus is already parasitized and meningoencephalitis is established [19], [44]–[47]. On the other hand, a higher parasitemia may be required as a prerequisite for CNS entry to initiate the neurological signs associated with Stage-2 HAT. Nonetheless, the finding that motile dividing extravascular *Tbb-O* and *Tbr*-T BSF were detectable for at least 3 days post infection (data not shown) suggests that the parasites can both cross the BBB and possibly survive in the brain parenchyma. In support of our finding, Stoppini and colleagues described the use of a murine organotypic brain culture to study the intracerebral phase of *T. b. brucei-*mediated trypanosomiasis *in vitro* [48]. Remarkably, the parasites survived for several weeks around the periphery and within the nervous parenchyma, although the culture medium alone was toxic to the parasites. The same also holds true for *T. b. rhodesiense* IL1852 in human organotypic cerebral cortex culture (Grab, unpublished observation). Furthermore, dividing parasites after 3 weeks did not *per se* interfere with neuronal activity of CNS based on extracellular electrophysiological recordings [48].

Our finding of early brain invasion by *Tbb* GVR/35-PKH67 is potentially profound. While *Tbr*-T and *Tbb-O* cause acute infection identical to wild type parasites, the *T. b. brucei* GVR/35 strain establishes a chronic CNS infection in mice with survival rates of up to 35 days [49]. Based on the degree of neuropathological damage, the level of clinical disability as quantified by reliable grading scales [50], [51], and magnetic resonance imaging [52], the *T. b. brucei* GVR/35 induced chronic murine disease reproduces features typically associated with CNS HAT [4]. Recent experiments conducted at the University of York have shown *T. b. brucei* GVR/35 BSF in murine brain sections by day 2 post infection (Lorna MacLean, personal communication), and studies conducted at the University of Glasgow show GVR/35 BSF in the murine brain on day 5 post infection by two-photon microscopy and on day 8 by bioluminescence imaging with an IVIS system (Elmarie Myburgh, personal communication). Most importantly, some Tbr-infected patients in the Soroti region of Uganda presented with an early onset of neurodysfunction during Stage-1 HAT [53]. Together with these findings, our data challenge the current paradigm that *T. b. brucei* GVR/35 BSF invade the CNS only between 14–21 days, because mice can be cured with the non-CNS drug Berenil if treated within 14–21 days, i.e. before neurological involvement occurs, but not if treated later than 21 days after infection when Berenil is no longer curative [4], [5], [52].

The cerebral microvascular system contains two functionally distinct barriers, the physiological BBB, which is formed by capillaries and serves as a tight diffusion barrier for solutes, and the neuroimmunological BBB, which allows diapedesis of immune cells and plays a unique role in neuroinflammation [28]. Our IVM observations show that extravascular trypanosomes are predominantly associated with PCVs rather than capillaries or arterioles, thus validating previous reports [2]. While our current studies do not reveal the exact route of the parasites to this location, our hypothesis is that trypanosomes rapidly cross the neuroimmunological BBB and enter the PVS [54]. However, until this event is documented in real time by further study, alternative possibilities must be considered. For example, some parasites may conceivably reach the PVS by following the flow of the CSF from the choroid plexus. While often considered a hostile environment for trypanosomes [11], [14], [19], CSF from Stage-1 and Stage-2 HAT patients supports the survival of *T. b. brucei* for 20 and 10 hours, respectively [55]. This window provides ample time for the parasites to travel inside the PVS from the choroid plexus all the way down to the level of PCVs [13], [15]. IVM studies in combination with static microscopic examination are currently underway to distinguish between these different scenarios.

The cortical microvasculature of trypanosome-infected mice exhibited arrested mononuclear leukocytes, whose number appeared to increase with disease progression. Leukocyte arrest was observed in PCVs and capillaries, but not in arterioles, and occasionally resulted in microvascular occlusion and focal interruption of the blood flow. Which leukocyte subpopulation is associated with microvascular inflammation and/or entry into the PVS and brain parenchyma remains to be determined. Leukocyte accumulation was accompanied by vascular leakage into the surrounding brain parenchyma, the extent of which appeared to correlate with size of parasite inoculum and time post infection. While there was no evidence for BBB impairment 5 h post infection with 10^6^ *Tbr*-T or 10^6^ *Tbb* GVR/35-PKH67 BSF, 24 h post infection with 10^5^ or 10^6^ *Tbb-O* BSF, or 48 h post infection with 10^5^ *Tbb-O* BSF, leakage of fluorescent BSA into the parenchyma was seen 48 h after inoculation of 10^6^ *Tbb-O* BSF, i.e. at the time of leukocyte recruitment to PCV. Interestingly, different neurotropic microorganisms possess unique mechanisms for interaction with the BBB [25]. For example, while African trypanosomes appear to traverse the BBB without inflicting significant damage [21], the strictly intravascular malaria parasite *Plasmodium* can induce severe microvascular inflammation and coagulation resulting in neurological impairment, coma, and death [25], [56]–[58]. Using a murine model of cerebral malaria and IVM, we found that neurological signs are closely correlated with the recruitment of various immune cell populations to PCVs and widespread disruption of the neuroimmunological BBB [27]. While the immune response to trypanosome infection awaits further characterization, our data demonstrate that both *T. b. brucei* and *T. b. rhodesiense* enter the brain parenchyma within hours, long before disruption of the BBB is apparent. Interestingly, trypanosomes were also found in the cerebrospinal fluid of monkeys early after infection when Stage-1 drugs were still effective [59].

Many questions still remain and further clarification of parasite interactions with the BBB endothelium and underlying CNS components is needed. Trypanosome passage across the murine BBB is a complex process that depends on many biological variables related to parasite subspecies and host genetics [60]. Is the ability to transmigrate across the BBB early an inherent property of a single parasite, or does the passage across the BBB require the interaction from a quorum of parasites? It is also possible that a large parasite load needs to invade the brain to produce a CNS infection significant enough to produce a CSF pleocytosis. Perhaps a few parasites manage to enter the brain during the early stage of infection, but it may take a week or two for a sufficiently large parasite load to produce CNS disease. Further, *in vitro* models suggest that trypanosome traversal across the BBB may occur bidirectionally [61], which is supported by the relapses observed in animals after treatment with non-CNS drugs such as Berenil [62]–[64]. For our data to be clinically important, one would expect the parasites to persist in the brain parenchyma. Considering the dense vascularization of the brain with a mean intercapillary distance of only 40 µm [65], extravascular trypanosomes can readily be tracked for long periods of time using a chronic model for brain IVM [66] in combination with trypanocidal drugs for elimination of intravascular parasites. Parasites that are inaccessible to IVM analysis because they are located in deeper layers of the cortex or the white matter, in the cerebellum or the brain stem, can be monitored with conventional techniques or *ex vivo* in live tissue sections.

While our findings have implications for both animal models and human disease, the only current way to correlate data from mouse models with those from HAT patients is to detect trypanosomes and/or white blood cells in the CSF at Stage-2 of the disease. Because there are presently no methods to directly visualize trypanosomes in the human CNS, the complex mechanisms associated with the pathogenesis of HAT are still poorly understood. It is currently impossible to distinguish Stage-1 from Stage-2 disease on the basis of clinical findings alone and CSF analysis remains the only accepted method to diagnose CNS infection. Interestingly, neurological features were recently reported in sleeping sickness patients with Stage-1 disease [53]. Although the possibility cannot be excluded that systemic infection can induce CNS symptoms, this study supports our hypothesis in that trypanosomes may enter the brain during early stages of the infection and cause mild early neurological symptoms, but that protective IL-6 and IL-10 mediated immune responses of the host prevent major damage to the CNS until later stages of the disease [67]. Our knowledge of the early pathogenesis of HAT, which includes the role of host genetics (i.e. rodent strain) in brain susceptibility to early trypanosomes entry, is still in its infancy. However, the presence of trypanosomes in the brain hours after infection may be supportive of treating very early Stage-2 disease with early stage drugs, a notion that has been suggested as constituting an intermediate stage of infection [3]. As this is the first IVM demonstration of trypanosomes in the brain parenchyma as early as hours post infection, the significance of this finding to human disease and the use of Stage-1 drugs must await the results of future clinical trials including assessment of whether current Stage-1 drugs have any brain penetration and/or allow/bolster neuroimmune systems to protect the brain early in disease. Nevertheless, the observations reported here suggest that some case failures of Stage-1 drugs may be due to early access to brain parenchyma by trypanosomes.

During these studies, several precautions were taken to ascertain that pathophysiological findings could be distinguished from methodological artifact. Data acquisition based on confocal Z-stacks through the 10–15 µm thick meninges in astrocyte-rich areas showed that microscopic imaging was possible to a depth of approximately 50 µm. Thus, it was clear that confocal microscopy would be a suitable methodology to examine in real time, live extravascular trypanosomes in the cortical microvasculature. Furthermore, intravital brain microscopy using over one hundred mice infected with brain tropic-pathogens that include *Trypanosoma brucei* (this study), murine-infective *Plasmodium* (Movila, Nacer and Frevert, manuscript in preparation, and [27]) and *Borrelia burgdorferi* (Movila and Frevert, unpublished) has enabled us to distinguish between pathophysiological disease-associated vascular alterations and surgery-induced artifacts. Mice with damage to the pial or cortical vasculature were excluded from the study. Optimization of excitation conditions under reduced laser power minimized phototoxicity and bleaching. In addition, precautions implemented to avoid surgery related-artifacts included i) imaging of microvessels only at the center of the cranial window to avoid possible artifacts along the perimeter of the craniotomy, ii) exclusion of pial microvessels in close proximity to the cranial coverslip, iii) stabilization of body temperature, and iv) minimization of respiration-induced movement of the brain. Using these procedures, none of the *Trypanosoma*-infected mice exhibited evidence for neurological impairment prior to surgery and imaging [30]. Finally, perhaps the most important confirmation that parasite entry into the brain parenchyma is not simply a surgery-induced artifact in the preferential localization of the parasites near PCVs, a finding validating previous reports concerning the site of trypanosome entry into the brain [2].

By establishing a method for long-term IVM analysis of CNS trypanosomiasis through a cranial window, we have set the stage for future functional studies with molecular markers, knockout mice, transgenic parasites, and RNAi. These studies will provide a better understanding of how intracellular signaling events allow trypanosome entry into the parenchyma and how endothelial activation, induction of inflammatory processes, cytokine secretion, leukocyte recruitment, and BBB alterations are involved in disease progression. A better understanding of parasite entry into the CNS, whether individually or as a group, may lead to novel therapeutic approaches and targets for treatment of neurological dysfunction.

## Materials and Methods

### Ethics Statement

This study was conducted in strict accordance with the recommendations in the Guide for the Care and Use of Laboratory Animals of the National Institutes of Health. The protocol was approved by the Institutional Animal Care and Use Committee of NYU School of Medicine (protocol number 100651). All surgery was performed under ketamine/xylazine/acepromazine anesthesia and all efforts were made to minimize suffering.

### Trypanosomes

For our *in vivo* model of experimental CNS infection we chose 1) the human CSF isolate *T. b. rhodesiense* IL 1852 [68], 2) *T. b. brucei* 90-13 bloodstream forms (BSF) [69], a strain previously used to demonstrate the possible role for brucipain in facilitating trypanosome entry into the brain [26], and 3) *T. b. brucei* GVR/35, a strain that causes a chronic CNS disease in mice [4]. For visualization by IVM, *T. b. brucei* GVR/35 BSF were fluorescently tagged with the cell tracker dye PKH67 (Sigma, St. Louis, MO) [21] and named *Tbb* GVR-PKH67 here. Parasites were maintained in mice and readily adapt to *in vitro* culture [70].

### Construction of Plasmids Encoding Fluorescent Proteins

*T. b. brucei* 90-13 were engineered to express monomeric mOrange (*Tbb*-O) and *T. b. rhodesiense* IL 1852 BSF to expressing tandem dimeric tdTomato (*Tbr*-T). The phD1034 vector and the plasmids pRSETOrange and tdTomato were gifts from Oliver Balmer [71] and Roger Tsien [72], respectively. The open reading frame for each fluorescent marker was amplified by PCR using the following primers:

**mOrange/eGFP fwd**: 5′-aagcttATGGTGAGCAAGGGCGAGGAG-3′

**mOrange/eGFP rev**: 5′-ggatccTTACTTGTACAGCTCGTCCAT-3′

**tdTomato fwd**: 5′-cagatatctaagcttATGGTGAGCAAGGGCGAGGAG-3′

**tdTomato rev**: 5′-cagagactaggatccTTACTTGTACAGCTCGTCCCA-3′.

The amplified open reading frames for each fluorescent marker were then sub-cloned into the pHD1034 vector at the *HinDIII/BamHI* sites. Each recombinant plasmid was propagated and amplified in *E. coli.*

### Trypanosome Transfection

Ten µg of pHD1034 vector containing cDNAs for each fluorescent marker was linearized with *NotI* restriction endonuclease and precipitated with ethanol. The DNA was re-suspended in 10 µl of H_2_O and mixed with a 100 µL suspension of *T. brucei* in Nucleofector T Cell solution (Lonza Inc. Allendale NJ). For electroporation, 10^7^ parasites were pelleted by centrifugation. The parasites were pulsed with the Nucleofector program×001. After pulsing, the parasites were transferred to 24 mL of complete medium and incubated overnight at 37°C with 5% CO_2_. To select for parasites stably expressing the fluorescent proteins, puromycin was added to the parasites at a concentration of 0.2 mg/ml and the parasites were aliquoted in a 24 well plate for 7–12 days. Stably transfected trypanosomes confirmed by fluorescent microscopy and flow cytometry for these studies were maintained in normal HMI-9 medium. Both *Tbr*-T and *Tbb*-O were confirmed to cause acute infection in mice similar to wild type parasites and behave like their respective wild type parental strains in acute mouse models [26]. For example, mice inoculated with 250 fluorescent *Tbr* show parasitemia within 6 days post infection and succumb to the disease within 21 days and mice infected with 600 *Tbb*-O begin to die 13 days after infection (data not shown).

### Mice and Infection

Four month-old female C57Bl/6 mice (Taconic Farms) were inoculated into the retroorbital sinus or intraperitoneally with 10^5^ or 10^6^ culture-derived fluorescent *Tbr*-T, *Tbb*-O, or *Tbb* GVR/35-PHK67 BSF as indicated for the individual experiments. Mice were housed in a parasite-free environment on a 12 h light/dark program and maintained and used in accordance with recommendations in the guide for the Care and Use of Laboratory Animals.

### Anesthesia and Craniotomy

At selected times post infection, mice were anesthetized by intraperitoneal injection of a cocktail of 50 mg/kg ketamine (Ketaset, Fort Dodge Animal Health, Fort Dodge, IO), 10 mg/kg xylazine (Rompun, Bayer, Shawnee Mission, KS), and 1.7 mg/kg acepromazine (Boehringer Ingelheim Vetmedica, St. Joseph, MO) (KXA mix) as described [73], [74] and surgically prepared for intravital imaging of the brain [75]. After immobilization of the head in a stereotaxic apparatus (mouse and neonatal rat adaptor; Stoelting, Wood Dale, IL), the skull was exposed by midline incision of the skin and a cranial window 4–5 mm in diameter was generated using a high-speed microdrill (Fine Science Tools, Foster City, CA). After removal of the bone flap, the exposed Dura mater was kept moist with gelatin foam (Gelfoam, Pfizer Inc., New York) until the window was closed with a cover slip glued to the skull [76]. Placement of the cranial window over the parietal cortex allowed imaging of cortical branches of the anterior cerebral artery, smaller arterioles down to capillary size, PCV, and superficial parietal veins that drain into the superior sagittal sinus.

### Intravital Microscopy

The cortical microvasculature was imaged with an inverted Leica TCS SP2 AOBS confocal system [73], [74]. Microvessels at the center of the cranial window were chosen for imaging to avoid possible surgery-related artifacts along the perimeter of the craniotomy. Meningeal vessels, which are readily identifiable by their location on the very surface, i.e. in direct contact with the coverslip, were excluded from the study. Respiration-induced movement of the brain was minimized by immobilization of the cover slip on a custom-made stainless steel adapter for the microscope stage. The body temperature of the animals was stabilized with a temperature-controlled Ludin chamber attached to the microscope. Periodic subcutaneous reinjection of KXA mix allowed intravital microscopic examination of the brain for 1–2 hours as in the past with *Plasmodium*-infected mice [27]. To visualize nuclei and the vascular lumen, mice were injected into the retroorbital sinus with 150 µl of a mixture of 1–2 µg/ml of the membrane-permeable DNA stain Hoechst 33342 and 5 µg/ml Alexa 647-conjugated bovine serum albumin (BSA) (Invitrogen, Carlsbad, CA), respectively. Appropriate laser lines were used to excite Hoechst (405 nm), tdTomato (543 nm), mOrange (543 nm), and Alexa 647 (633 nm). Laser power was reduced to a minimum to minimize phototoxicity and bleaching. Optimized excitation conditions have allowed us to monitor live fluorescent parasites for a period of up to 6 h without any apparent effect on viability [73], [74].

Intravital brain microscopy of more than one hundred mice, conducted in the course of the studies presented here, IVM of experimental cerebral malaria (Movila, Nacer and Frevert, manuscript in preparation, and [27]) and neuroborreliosis (Movila and Frevert, unpublished), has enabled us to distinguish between disease-associated vascular alterations and surgery-induced artifacts. Mice with damage to the pial or cortical vasculature typically exhibit extensive bleeding and profuse leakage of the fluorescent vascular marker. Such animals do not produce useful data and were excluded from the study.

### Vibratome Sectioning

Vibratome sections of unfixed brain tissue from infected mice that had been injected with fluorescent markers were used to facilitate parasite detection at early times post infection. Thick sections (100–200 µm) were cut with a Leica VT1200 Semiautomatic Vibrating Blade Microtome (Leica Microsystems, Bannockburn, IL) and imaged immediately after transfer into medium.

### Image Processing

Multiple confocal time series of the cortical microvasculature were acquired with Leica Confocal Software. Imaris 7.4 and Imaris Track (Bitplane, Saint Paul, MN), Image-Pro Plus (Media Cybernetics, Bethesda, MD), AutoDeBlur (Media Cybernetics, Bethesda, MD), and NIH ImageJ were used for deconvolution and analysis of time sequences.

## Supporting Information

Movie S1 **Trypanosomes enter the brain parenchyma.** One day post infection with 10^6^ *Tbb-O*, a motile *Tbb-O* BSF (red) is located adjacent to a PCV. The vascular lumen is visualized with Alexa 647-conjugated BSA (shown in green). RBCs in the lumen of the PCV exclude the vascular maker and appear as negatively stained (dark) streaks. Nuclei are blue.
(AVI)

Movie S2 **Trypanosomes divide within the brain parenchyma.** DNA staining reveals two nuclei (large Hoechst-stained organelles) and two kinetoplasts (small Hoechst-stained organelles) within a motile extravascular BSF (red) 24 h post infection with 10^6^ *Tbb-O*. The vascular lumen is green, nuclei are blue.
(AVI)

Movie S3 **Trypanosomes travel with the flagellar tip leading.** Temporary leukocyte-mediated interruption of the blood flow allows a *Tbb-O* (red) to swim back and forth in a capillary 48 h post infection with 10^6^ BSF.
(AVI)

Movie S4 **Extravascular trypanosomes and leukocyte recruitment to PCVs.** Two days post infection with 10^6^ *Tbb-O* BSF, motile BSF are located in the brain parenchyma adjacent to a PCV. Large numbers of red streaks in the vascular lumen indicate high parasitemia. A leukocyte adheres to the wall of the PCV.
(AVI)

Movie S5 **Leukocyte recruitment and vascular leakage.** Two days post infection with 10^6^ *Tbb-O* BSF, a cluster of extravascular BSF is located in the PVS (lower left), while many other BSF have entered the parenchyma (upper right). The vascular marker BSA-Alexa 647 (shown in green) has leaked into the PVS. The PCV contains arrested leukocytes (dark cells). The narrow dark streaks in the center of the PCV represent fast-moving blood cells demonstrating unimpaired blood flow.
(AVI)

Movie S6 **Leukocyte recruitment and vascular occlusion.** Two days after inoculation of 10^5^ *Tbb-O*, a motile BSF (red) is located in the brain parenchyma near a PCV. While numerous other trypanosomes travel at bloodstream velocity in the PCV, the flow in a neighboring capillary (top) is impaired by arrested leukocytes.
(AVI)

Movie S7 **Leukocyte recruitment and capillary occlusion.** An arrested mononuclear leukocyte occludes a capillary and diverts the blood flow into a collateral microvessel. *Tbb-O* BSF (red) become distorted when moving at high velocity. The vascular lumen is green, nuclei are blue.
(AVI)

Movie S8 **Leukocyte recruitment after low-dose infection.** Intravascular *Tbb-O* and arrested mononuclear leukocytes are rare 24 h post infection with 10^5^ BSF.
(AVI)

Movie S9 **Leukocyte recruitment after prolonged low-dose infection.** The number of arrested mononuclear leukocytes has increased between 24 h and 48 h post infection with 10^5^ *Tbb-O*. Numerous red streaks in the lumen of the PCV indicate the higher level of parasitemia.
(AVI)

Movie S10 ***Tbr*** **-T BSF in the parenchyma 5 hours post infection.** A motile *Tbr*-T BSF (red) is located in the brain parenchyma adjacent to a PCV after infection with 10^6^ BSF. Note the absence of arrested leukocytes and leakage of the vascular marker (red) into the surrounding tissue. The vascular lumen is green, nuclei are blue.
(AVI)

Movie S11 ***Tbb*** **BSF in the parenchyma 5 hours post infection.** A motile *Tbb* GVR/35 BSF, labeled with PKH67 (green), can be seen next to a PCV. Intravascular parasites are rare. Note the absence of arrested leukocytes and leakage of the vascular marker (red) into the surrounding tissue. Nuclei are blue.
(AVI)
